# Retinal ganglion cell axonal compression by retinal vessels in light-induced retinal degeneration

**Published:** 2011-06-25

**Authors:** Diego García-Ayuso, Manuel Salinas-Navarro, Marta Agudo-Barriuso, Luis Alarcón-Martínez, Manuel Vidal-Sanz, María P. Villegas-Pérez

**Affiliations:** 1 Laboratorio de Oftalmología Experimental, Facultad de Medicina, Universidad de Murcia, Campus de Espinardo, 30100 Espinardo, Murcia, Spain; 2 Unidad de Investigación. Hospital Universitario Virgen de la Arrixaca. Servicio Murciano de Salud, Fundación para la Formación e Investigación Sanitarias de la Región de Murcia. 30120 El Palmar, Murcia, Spain

## Abstract

**Purpose:**

To analyze the damage produced by light in mydriatic and miotic albino retinas under two different sources of light.

**Methods:**

Albino Sprague Dawley female rats were exposed to 3,000 lx during 48 h under two different light sources: linear and circular bulbs. Before exposure, their left pupils were dilated. Before and at different times after light exposure (ALE), electroretinographic signals were recorded. One week before processing, retinal ganglion cells (RGCs) were traced by applying fluorogold on the superior colliculi. Just before processing, some animals were intravenously injected with horseradish peroxidase to analyze retinal vascular leakage. At different times ALE, animals were sacrificed and their retinas dissected as whole mounts or cross-sections. Cross-sections were used to study the retinal degeneration and to detect apoptotic nuclei by the transferase dUTP nick end labeling (TUNEL) technique. Whole mounts were used to analyze vascular leakage; investigate the nerve fiber layer, identified by immunodetection of neurofilaments; and quantify the whole population of RGCs identified by fluorogold tracing and Brn3a immunodetection. With the quantitative data, detailed isodensity maps were generated to study the spatial loss of RGCs.

**Results:**

Phototoxicity causes an immediate and permanent abolishment of the electroretinographic response. Early ALE, photoreceptors degenerate by apoptosis and this death is more severe in mydriatic conditions and under circular bulbs. Photoreceptor loss starts in an arciform dorsomedial retinal area, but at 3 months ALE has spread to the whole retina and there are no differences related to either pupil dilation or light source. Three months ALE, RGC axons show distorted trajectories and abnormal expression of neurofilaments. Six months or more ALE, there is significant death of RGCs caused by axonal strangulation by displaced inner retinal vessels. Topography of the surviving RGCs shows that their loss is not uniform throughout the retina.

**Conclusions:**

Light damage to photoreceptors depends on pupil dilation and light source, but affects all retinal layers with time. These deteriorative events are also observed in light-induced and inherited retinal degenerations in pigmented animals, but occur differently. Thus, the role of ocular pigmentation and the etiology of photoreceptor degeneration on retinal remodelling deserve further investigation.

## Introduction

Light-induced retinal damage, i.e., phototoxicity, is a well established model of retinal degeneration. This model is often used to study the factors leading to photoreceptor death, assess the course of subsequent degenerative events occurring in the retina, and test neuroprotective therapies for oxidative stress. Importantly, this model also covers the essential characteristics of human age-related macular degeneration [1].

Photoreceptor death, triggered by inherited dystrophies [2-5] or light exposure [1,6,7], affects all retinal layers with time. At late stages, in the innermost layer, the retinal vessels overlying the nerve fiber layer drag, compress, and sever the retinal ganglion cell (RGC) axons, finally causing the death of these neurons [3-6]. Thus, RGC death in this model is not secondary to photoreceptor degeneration, but rather to retinal remodelling after light exposure [1]. RGC death is also a common feature in retinitis pigmentosa [8-11], but it is not yet known if this is due to a similar mechanism.

In pigmented nondystrophic Royal College of Surgeons (RCS) and Lister-Hooded rats after light exposure (ALE), the existence of an “arciform area” of vascular leakage in the superotemporal retina, which was the first and more severely affected retinal area, was documented for the first time [6,7]. This area corresponds to the region of increased susceptibility to light damage described by other authors in normal rats [1,12-16] and in transgenic rat models of retinitis pigmentosa [17].

Why there is an arciform area in the superotemporal region of the rat retina that is more sensitive to light is, at the moment, a question for debate. Our group has also described that, in the rat retina, the highest RGC densities are found in the superior retina along the nasotemporal axis; we have proposed that this area may represent the visual streak of this species [18-20]. Since the arciform area and the visual streak in the rat show a similar topography, this arciform area could be the result of preferential fixation.

The description of the light-sensitive arciform area and RGC axonal compression and death in light-induced and inherited retinal degeneration involved pigmented animals. Hence, the question arose as to whether these events were dependent upon ocular pigmentation. Therefore, in this study, we have investigated the damage produced by light in mydriatic and miotic albino retinas under two different sources of light. Specifically we have studied: i) the temporal course and retinal location of photoreceptor degeneration; ii) the retinal functionality after light exposure; iii) the occurrence of vascular leakage; iv) the late appearance of displaced retinal vessels; and v) the degeneration of the inner retina, quantifying and mapping topographically the loss of RGCs.

## Methods

### Animal handling

Albino Sprague Dawley (SD) female rats weighing 150 to 180 g (2 months ±1 week of age) were obtained from the breeding colony of the University of Murcia. Rats were housed in temperature- and light-controlled rooms with a 12 h:12 h light-dark cycle (light from 8 AM to 8 PM), and had food and water ad libitum. Light intensity within the cages ranged from 5 to 30 lux (scotopic to mesopic conditions). Animal manipulations were performed following the Spanish and European Union regulations for the use of animals in research and the Association for Research in Vision and Ophthalmology (ARVO) statement for the use of animals in ophthalmic and vision research. Moreover, adequate measures were taken to minimize pain or discomfort.

Surgical manipulations were performed under general anesthesia induced with an intraperitoneal injection of a mixture of ketamine (70 mg/kg, Ketolar®, Parke-Davies, S.L., Barcelona, Spain) and xylazine (10 mg/kg, Rompún®, Bayer, S.A., Barcelona, Spain). For recovery from anesthesia, rats were placed in their cages and an ointment containing tobramycin (Tobrex®, Ophthalmic unguent Alcon S.A., El Masnou, Barcelona, Spain) was applied on the cornea to prevent corneal desiccation. Animals were sacrificed by an intraperitoneal injection of an overdose of sodium pentobarbital (Dolethal Vetoquinol®, Especialidades Veterinarias, S.A., Alcobendas, Madrid, Spain).

### Light exposure

Before light exposure, the left pupil was dilated with a drop of 1% atropine (Colirio de atropina al 1%, Alcon S.A., El Masnou, Barcelona, Spain) to assess the effect of dilation on retinal phototoxicity. Each animal was placed individually in a 23×23 cm standard transparent animal cage with an upper removable metallic grid and the food and the water were placed in Petri dishes within the cage. Litter covered the bottom of the cage, which was overlaid by a metal grid to prevent the animals from burying their heads in the litter to hide from light.

In a previous study in pigmented rats, to induce photoreceptor degeneration, we dilated the left pupils and exposed the animals to 3,000 lux continuously for 72 h [6,7]. In this study, we tried the same paradigm in the albino rats, but the results were devastating. Thus, we used a reduced exposure period to 48 h.

Continuous light exposure during 48 h was performed under two types of cool fluorescent white light lamps: i) two linear bulbs, situated approximately 20 cm above the cages, and ii) three circular bulbs surrounding the cages. For both types of bulbs, light intensity, when measured in the center of the cages, was approximately 3,000 lux, while in other areas within the cage it was 3,000±100 lux. Because the moment of the day at which exposure is initiated influences the amount of retinal phototoxicity, light exposure always started between 10 and 12 AM. Left eye mydriasis was checked every 24 h, and when the animals did not show full left eye dilation, they received another drop of atropine.

### Electroretinography

Electroretinographic (ERG) recordings were obtained from a group of 12 animals before light exposure (baseline) and 1 and 7 days ALE, from a group of three experimental animals at 6 months ALE, and one control animal of a similar age. ERG recordings were performed as previously described [21,22]. Briefly, the retina was stimulated with light intensities ranging between 10^−5^ and 10^−4^ cd∙s·m^−2^ for the scotopic threshold response (STR), between 10^−4^ and 10^−2^ cd·s·m^−2^ for the rod response, and between 10^−2^ and 10^2^ cd·s·m^−2^ for the mixed (rod and cone) response. Analysis of the recordings was performed using the International Society for Clinical Electrophysiology of Vision normalization criteria. The STR was analyzed for each stimulus: Positive STR (pSTR) was measured from baseline to the “hill” of the positive deflection, approximately 110 ms from the flash onset, and negative STR (nSTR) was measured from baseline to the first “valley” after the pSTR, about 220 ms from the flash onset.

### Horseradish peroxidase injection

To label the retinal blood vessels, a solution (143 mg/ml in sterile saline) of type I horseradish peroxidase (HRP, 44 kDa, P 8125, 12,000 units; Sigma) was injected in the femoral vein 15 min before processing, as previously described [3,6,7]. Forty animals received HRP injections and were processed at 0 h (n=18), 1 week (n=9), 1 month (n=7), and 3 months (n=6) ALE.

### Retinal ganglion cell retrograde labeling from the superior colliculi

To trace RGCs in a retrograde fashion, fluorogold (FG) was applied onto both superior colliculi (SCi), the main retinorecipient area in rodents, one week before animal processing, following the previously described methods, which are standard in our laboratory [3,5-7,18,22-24]. In brief, after exposing the midbrain, a small pledget of gelatin sponge (Espongostan® Film, Ferrosan A/S, Denmark) soaked in saline containing 3% FG and 10% dimethilsulfoxide (DMSO), was applied over the entire surface of both SCi. Previous studies in control rats in our laboratory have shown that FG application to the SCi results in the labeling of 98.4% of the RGC population in SD rats [18]. FG was applied to 44 animals: 5 control aged-matched animals (postnatal day 625: P625; approximately 21 months old) and 48 experimental animals that were processed at 0 h (n=8), or 1 (n=6), 3 (n=7), 6 (n=7), 9 (n=10), and 12 months (n=10) ALE, respectively.

### Tissue processing

Both eyes of each animal were processed. Some eyes were used for cross-sections and others for retinal whole mounts.

### Whole-mount preparations

HRP-injected animals were sacrificed at 0 h (n=18), 1 week (n=9), 1 month (n=7) and 3 months (n=6) ALE. Both eyes were enucleated and immersed for 1 h in a solution of 4% paraformaldehyde in 0.1 M PBS. Later, the retinas were dissected as whole mounts by making four radial cuts in the superior, inferior, nasal, and temporal retinal quadrants. Retinal orientation was maintained by making the deepest radial cut in the superior retina. The retinas were postfixed for 1 h in the same fixative, washed, reacted for HRP demonstration using a modified Hanker-Yates technique [3,6,7,25], mounted on subbed slides vitreal side up, and covered with antifading mounting media containing 50% glycerol and 0.04% p-phenylenediamine in 0.1 M sodium carbonate buffer (pH 9.0 [26]).

FG-traced animals were perfused transcardially through the ascending aorta, first with saline and then with 4% paraformaldehyde in 0.1 M phosphate buffer (pH 7.4). The eyes were enucleated and the retinas were dissected as whole mounts, as described in the previous paragraph.

### Immunohistofluorescence

To study RGC axons, all FG-traced whole-mounted retinas were incubated with the monoclonal antibody RT-97 (Hybridoma Bank, University of Iowa, IA) [2,3,27-29]. This antibody detects the phosphorylated high molecular weight (200 and 145 kDa) subunits of the neurofilament triplet (phosphorylated High molecular weight Neurofilament subunit [pNFH] see [30-32]). For quantification analyses, in some FG-traced retinas (see results), RGCs were also identified by incubation with goat anti-Brn3a (C-20, Santa Cruz Biotechnology, Heidelberg, Germany), as previously reported [5,19,20].

Briefly, retinas were permeated in PBS 0.5% Triton X-100 by freezing them during 15 min at −70 °C, rinsed in new PBS 0.5% Triton and incubated overnight at 4 °C with the primary antibody diluted (1:1,000 for RT-97 and 1:100 for Brn3a) in blocking buffer (PBS, 2% Triton, 2% normal donkey serum, Jackson ImmunoResearch, Suffolk, UK). Then, retinas were washed three times in PBS and incubated for 2 h at room temperature (RT) with the secondary antibody (donkey Alexa-488 antimouse IgG(H^+^L) or donkey Alexa-594 antigoat IgG (H^+^L), Molecular Probes, Invitrogen, Barcelona, Spain), diluted 1:500 in blocking buffer. Finally, retinas were thoroughly washed in PBS and mounted vitreal side up on subbed slides and covered with antifading solution.

### Oriented cross-sections

#### Microtome sectioning

For this study, we used animals processed immediately (n=4) or 7 days (n=4), 1 (n=4), 3 (n=4), 6 (n=4), 9 (n=4), or 12 months (n=2) ALE. As control, two-month-old SD rats were used (n=4).

After perfusion (see above), the eyes were enucleated and the superior pole of the eye marked with china ink. After removing the cornea and lens, the eyecups were postfixed in the same fixative for 1 h and embedded in paraffin. Three micron thick sagittal sections were obtained in a microtome and stained with Hansen’s hematoxylin and eosin or processed for terminal deoxynucleotidyl transferase dUTP nick end labeling (TUNEL) to detect apoptotic nuclei. The hematoxylin and eosin–stained sections were mounted with DPX (BDH, VWR International Ltd., Poole, England) for observation in the light microscope. The TUNEL assay was performed according to the manufacturer’s protocol (FragEL™ DNA Fragmentation Detection Kit, Qiagen, Merck Bio, Nottingham, UK) with slight modifications as follows: Biotin-labeled DNA was detected by 2 h incubation at RT with avidin-Tetramethyl Rhodamine Iso-Thiocyanate (TRITC; Sigma-Aldrich, Madrid, Spain) diluted 1:500 in PBS containing 0.1% Triton. After washing, slides were mounted with antifading medium containing 4',6-diamidino-2-phenylindole (DAPI; VectaShield mounting medium with DAPI, Vector, Atom, Alicante, Spain) to counterstain all retinal nuclei.

#### Cryostat sectioning

For this study, we used two control and four experimental retinas processed 12 months ALE. Eye cups were dissected and oriented as above. Then, they were cryoprotected by immersion in 15% sucrose (Sigma, Alcobendas, Madrid, Spain) before embedding them, with the superior pole in a known position, in optimal cutting temperature compound (Sakura Finetek, Torrance, CA) for cryostat sectioning. Sections (15 μm thick) were blocked in 5% normal donkey serum (Jackson ImmunoResearch Inc., Suffolk, UK) in PBS 0.1% Triton-100. RGC axons and vessels were doubly detected by a 3 h incubation at RT with a mixture of the primary antibodies rabbit anti-NFH (H-100, Santa Cruz Biotechnologies, Heidelberg, Germany) and mouse anti-rat endothelial cell antigen (RECA; mouse anti-RECA-1, AbD Serotec, Dusseldorf, Germany) diluted in blocking buffer (1:50 for anti-NFH and 1:1,000 for anti-RECA-1). Secondary detection was performed by incubating the sections, during 1 h at RT, with Alexa Fluor-488 donkey antirabbit IgG (H^+^L) and Alexa Fluor-594 donkey antimouse IgG (H^+^L) (Molecular Probes, Invitrogen, Barcelona, Spain), each diluted 1:500 in PBS 0.1% Triton-100. Finally, sections were thoroughly washed and mounted with antifading mounting medium containing DAPI to counterstain the nuclei (Vectashield mounting medium with DAPI, Vector, Atom, Alicante, Spain). Sections were observed and photographed under a light (hematoxylin and eosin staining) or fluorescence (TUNEL signal or RECA+NFH immunodetection) microscope.

### Retinal image analysis

Retinal whole mounts were examined and photographed under a fluorescence microscope (Axioscop 2 Plus; Zeiss Mikroskopie, Jena, Germany) equipped with different filters. The microscope was also equipped with a high-resolution digital camera (ProgRes^TM^ C10, Jenoptik, Jena, Germany), a computer-driven motorized stage (Pro-Scan^TM^ H128 Series, Prior Scientific Instruments, Cambridge, UK) controlled by IPP (IPP 5.1 for Windows^®^; Media Cybernetics, Silver Spring, MD) with a microscope controller module (Scope-Pro^®^ 5.0 for Windows^®^; Media Cybernetics, Silver Spring, MD), following standard procedures in our laboratory [5,18,19,24,33,34]. To reconstruct the retinal whole-mounts, retinal multiframe acquisitions were acquired in a raster scan pattern using a 10× objective (Plan-Neofluar, 10×/0.30; Zeiss Mikroskopie, Jena, Germany). Single frames were focused manually before the capture of the digitized images. Because the frame size was 0.627 mm^2^/image, we usually needed to acquire 154 images to scan the entire retina.

The images taken for each retina were saved as a set of 24-bit color image pictures and later, these images were combined into a single high-resolution composite image of the whole retina using IPP. Reconstructed images were further processed using Adobe Photoshop® CS 8.0.1 (Adobe Systems, Inc., San Jose, CA) when needed.

### Retinal ganglion cell counting

The individual FG or Brn3a fluorescent images taken in each retina were processed by a specific cell counting subroutine developed by our group. Briefly, we used the IPP macro language to apply a sequence of filters and transformations to each image to clarify cell limits and separate individual cells for automatic cell counting. This procedure has been previously reported in detail [18,19,24].

### Isodensity maps

To demonstrate the spatial distribution of FG and Brn3a positive RGCs, isodensity maps were generated using the specific subroutine developed in our laboratory [5,18,19,24]. Briefly, using the IPP macro language, every frame was divided into 64 (FG) or 36 (Brn3a) sampling areas, in which RGC counts were obtained and cell densities calculated. These densities were represented as filled contour plots using the graphics software SigmaPlot^®^ for Windows^TM^ (Version 8.0; SPSS, Inc.).

### Statistics

Statistical analysis was done using SigmaStat^®^ 3.1 for Windows^®^ (SigmaStat^®^ for Windows^TM^ Version 3.11; Systat Software, Inc., Richmond, CA).

### Electroretinographic data

The paired *t* test was used to compare electroretinogram wave amplitudes before and after light damage in the same animals (0–1 month ALE). The groups analyzed 3 and 6 months ALE were compared to age-matched animals using the unpaired *t* test. The percent wave amplitude response ALE was calculated using the values obtained before light exposure. The Mann–Whitney test was used to compare amplitude percentages between 1 day and 7 days ALE.

### Retinal ganglion cell population

The Kruskal–Wallis test was used to compare more than two groups, and the Mann–Whitney test was used when comparing two groups only. Differences were considered significant when p<0.05.

## Results

### Light exposure abolishes retinal activity

As a baseline, the ERG signal was recorded simultaneously from both eyes before light exposure.

**Figure 1:**
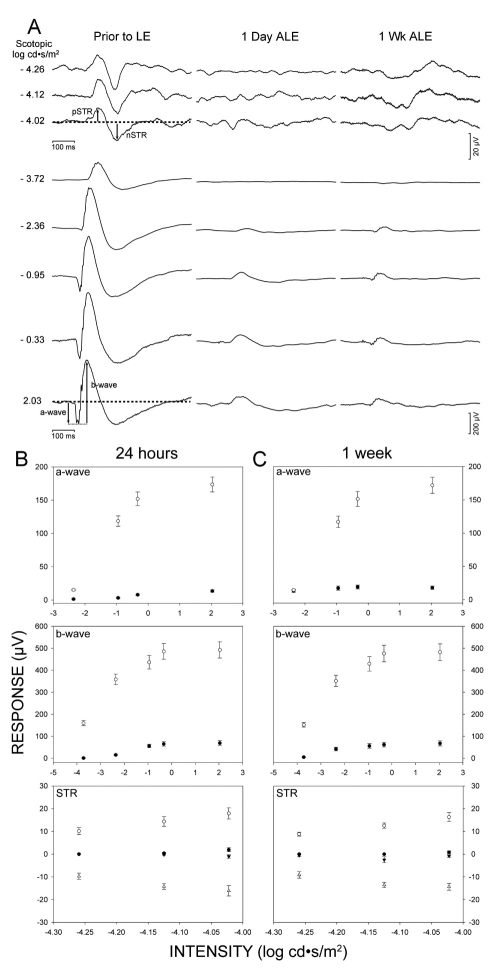
Light exposure abolishes the electroretinographic response. **A**: Electroretinograms from the left eyes of albino rats (n=12), before light exposure and 1 day and 1 week after light exposure (ALE). **B**-**C:** Mean amplitudes (±SE) of the a- and b-waves and scotopic threshold response (STR) before light exposure (open symbols) and 24 h (**B**) and 1 week (**C**) ALE (closed symbols). Error bars are shown when larger than symbols.

One day ALE (n=12), the scotopic response had almost disappeared from both eyes (Figure 1A), with a significant decrease (paired *t* test; p<0.001) of the pSTR and nSTR waves down to approximately, 6% and 3% of the control values, which were arbitrarily considered 100% (Figure 1B). The scotopic and mixed responses in the light-exposed animals also showed a significant decrease of the a- and b-waves (paired *t* test; p<0.001) as they were reduced, approximately, to 10% (Figure 1B) of the control values.

One week ALE, the scotopic and mixed responses were similar and not significantly different (Mann–Whitney test, p>0.05) to those found 1 day ALE (Figures 1A,C). The a-wave showed a partial recovery for the dimmest stimulus but remained at the levels found 1 day ALE for the rest of the stimuli (compare Figure 1B,C). Six months ALE, the ERG response was normal in the control age-matching animal and completely abolished in the experimental animals (n=3, data not shown).

In conclusion, the ERG data indicate that there was an almost complete abolition of the retinal activity from the first day ALE that persisted for the duration of the study (6 months).

### Light-induced vascular leakage: the arciform area

**Figure 2:**
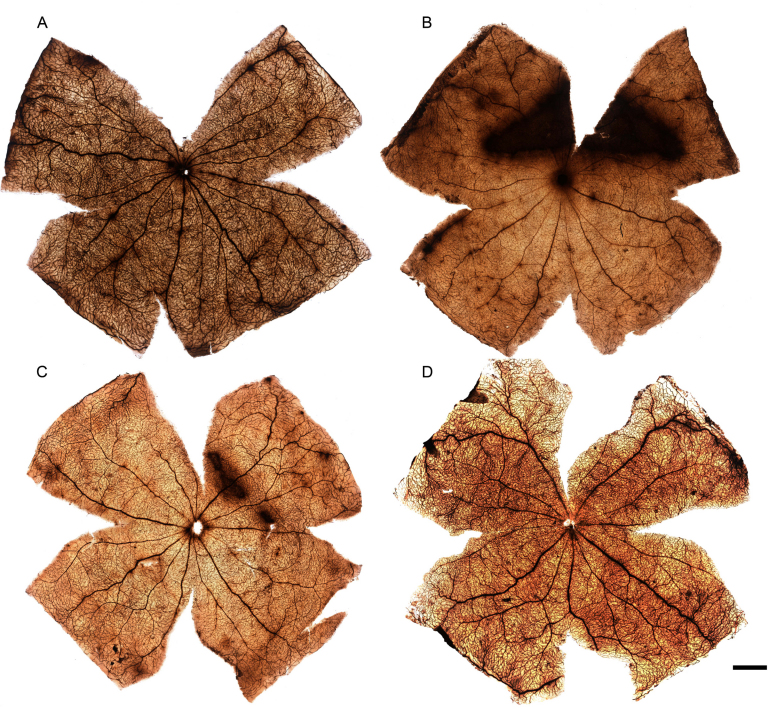
Light exposure provokes retinal vascular leakage. Photomontages of the retinal whole mounts of one control (**A**) and three light-exposed animals (**B**, **C**, **D**) processed 0 h (**B**) or 7 (**C**) or 30 days (**D**) after light exposure (ALE). The retinal vessels appear labeled with horseradish peroxidase (HRP) in all the retinas and there is an “arciform area” showing HRP leakage in the retinas of the animals processed 0 h (**B**) and 7 days (**C**) ALE. The scale bar represents 1 mm.

HRP labeling in whole-mounted retinas was performed to investigate vascular leakage in the photoexposed retinas. Diffuse HRP leakage was found at 0 (n=18 rats, i.e., 18 left and 18 right retinas) and 7 days (n=9 rats) ALE in the previously described arciform area of the superotemporal retina (Figure 2B,C), but not at longer times (1 [n=7 rats] and 3 [n=6 rats] months). The size of the arciform area showing HRP leakage varied between animals, but it was usually greater in the retinas processed at 0 h than at 7 days ALE.

HRP leakage occurred in dilated and nondilated eyes, but it was more frequent in the dilated left eyes (Figure 2B,C). Out of all the retinas analyzed at 0 days ALE, vascular leakage was observed in 13 left (72%) and 3 right (23%) retinas. This proportion diminished with time and at 7 days, only three left and three right retinas (18%) showed vascular leakage in the arciform area. In conclusion, in the albino rat, HRP leakage is found more frequently in the left (dilated) eyes than in the right (nondilated) eyes, and this leakage occurs very early after ALE.

### Time course of light-induced photoreceptor degeneration: effect of the light source and pupil dilation

**Figure 3:**
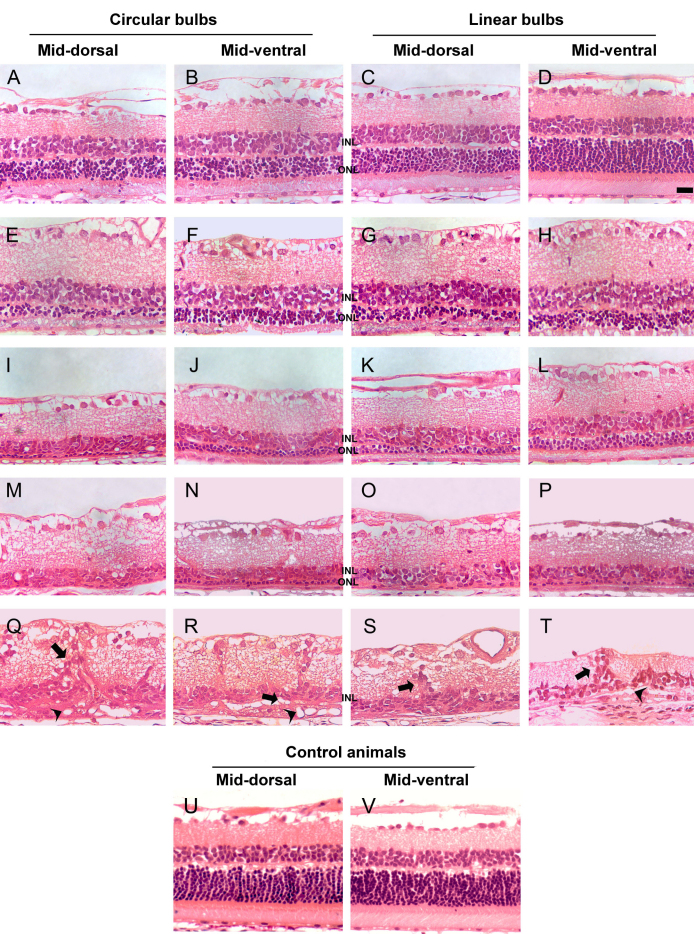
Temporal and spatial course of photoreceptor loss after light exposure. Hematoxylin and eosin–stained retinal cross-sections of control retinas (lower row) and of the left (dilated) eyes from animals photoexposed under circular (left two columns) or linear (right two columns) bulbs. Images were taken from the mid-dorsal and midventral retina. **A-D**: animals processed at 0 h, **E-H**: 7 days, **I-L**: 1 month, **M-P**: 3 months, **Q**: 6 months, **R**, **S**: 9 months, **T**: 12 months after light exposure (ALE) and control animals. **U**-**V** represents control animals. Photoreceptor loss is observed in all sections but in **D** (animals processed 0 h ALE; ventral retina), where the retinal structure is conserved and is similar to control animals. Photoreceptor loss was always more severe in the dorsal retina. During the first 3 months ALE, retinal damage was more drastic in the animals exposed to circular bulbs; however, from this time point onwards, it was similar in all animals (**M-P**). Six or more months ALE (**Q**-**T**), vascular complexes (arrowheads) were observed in the subretinal space, sometimes connected to vessels that ran vertically in the retina and that are surrounded by nonpigmented cells (arrows). The scale bar represents 100 µm.

Control animals showed an outer nuclear layer (ONL) 12 to 14 nuclei thick and an inner nuclear layer (INL) 3 to 4 nuclei thick (Figure 3U-V; Table 1). The experimental retinas showed a progressive photoreceptor loss that was evident as soon as 0 days ALE (Figure 3, Table 1). Independently of pupil dilation or the light source, this loss was always more severe in the superior mid-dorsal retina, a location that corresponds to the arciform area of HRP leakage (Figure 3). Nonetheless, both the dorsal and ventral retinas were gravely affected and only the extreme periphery was intact; because the retina is thinner there, a transition zone was never seen. Pupil dilation induced an earlier and quicker photoreceptor loss but, by 1 month ALE, retinal degeneration had reached similar levels in both eyes (Table 1). Circular bulbs (Figure 3, first and second columns) induced more severe loss than linear bulbs (Figure 3, third and fourth columns), but only up to the third month ALE (Table 1 and Figure 3, compare panel A-B and panel E-F with panel C-D and panel G-H, respectively), and by this time or more ALE, the appearance of the retina was similar in both experimental groups (Figure 3M-T; Table 1).

**Table 1:** Temporal course of retinal degeneration after light exposure: effect of eye pigmentation, light source and pupil dilation

**Time ALE**	**Pigmented rats**				**Albino Sprague-Dawley rats**							
**(Data from: [**6**])**				**3000 lx during 48 h**							
**3000 l× during 72 h**				**(n=26)**							
**Linear Bulbs**				**Circular Bulbs (n=13)**				**Linear Bulbs (n=13)**			
**Dorsal retina**		**Ventral retina**		**Dorsal retina**		**Ventral retina**		**Dorsal retina**		**Ventral retina**	
**Left eye (dilated)**	**Right eye (undilated)**	**Left eye (dilated)**	**Right eye (undilated)**	**Left eye (dilated)**	**Right eye (undilated)**	**Left eye (dilated)**	**Right eye (undilated)**	**Left eye (dilated)**	**Right eye (undilated)**	**Left eye (dilated)**	**Right eye (undilated)**
0 h	0–1	10–12	2–4	10–12	4–6	5–7	6–8	10–12	5–7	6–8	10–12	10–12
					(n=2)				(n=2)			
1 week	0–1	10–12	2–4	10–12	0–1	1–2	2–4	3–5	1–2	1–3	3–5	3–6
					(n=2)				(n=2)			
1 month	Reduced OPL. No ONL	10–12	Reduced ONL and OPL	10–12	Reduced OPL. No ONL.		Reduced OPL and ONL (1–3 nuclei).		Reduced OPL and ONL (0–2 nuclei).		Reduced OPL and ONL (1–3 nuclei).	
					(n=2)				(n=2)			
3 months	No ONL. No OPL. Reduced INL (0–1 nuclei).	10–12	Reduced ONL(0–1 nuclei).	10–12	No ONL. No OPL. Thinner INL (2–3 nuclei). (n=4)							
6 months or longer	Very thin retina, no layers. Subretinal vessels. RGC loss.	10–12	Reduced ONL (0–1 nuclei). Thinner INL (1–3 nuclei). Subretinal vessels. RGC loss.	10–12	Very thin INL (1–2 nuclei). Subretinal complexes of vessels. Significant RGC loss. (n=10)							

Data from the first column correspond to pigmented Lister-Hooded and pigmented non-dystrophic Royal College of Surgeons rats exposed to 3,000 lx during 72 h from a previous study (Marco-Gomariz et al., 2006, reference 6). Control animals in this and the previous study and the undilated right eyes of pigmented rats in the previous study showed an outer nuclear layer (ONL) 12 to 14 nuclei thick and an inner nuclear layer (INL) 3 to 4 nuclei thick (not shown). Numbers in 0 h and 1 week rows and in all rows of the right undilated eyes of the pigmented animals refer to the nuclei thickness of the ONL. n=number of animals. ONL: outer nuclear layer, OPL: outer plexiform layer, INL: inner nuclear layer, GCL: ganglion cell layer, NFL: nerve fiber layer. RGC: retinal ganglion cells.

Six months or more ALE, the degree of retinal disorganization had progressed further: The ONL had disappeared, leaving only a very thin INL and the inner plexiform, ganglion cell, and nerve fiber layers (Table 1, Figure 3Q-T). After this time point, tortuous complexes of vessels appeared subretinally (between the RPE cells and Bruch’s membrane) in the experimental retinas (Figure 3Q-T, arrowheads). These vessels were sometimes connected with vessels that ran vertically from the inner retinal vascular plexus and that were usually surrounded by nonpigmented RPE cells, which migrated along the surface of these vessels (Figure 3Q-T, arrows). This is further analyzed below.

### Light-induced photoreceptor death is apoptotic

**Figure 4:**
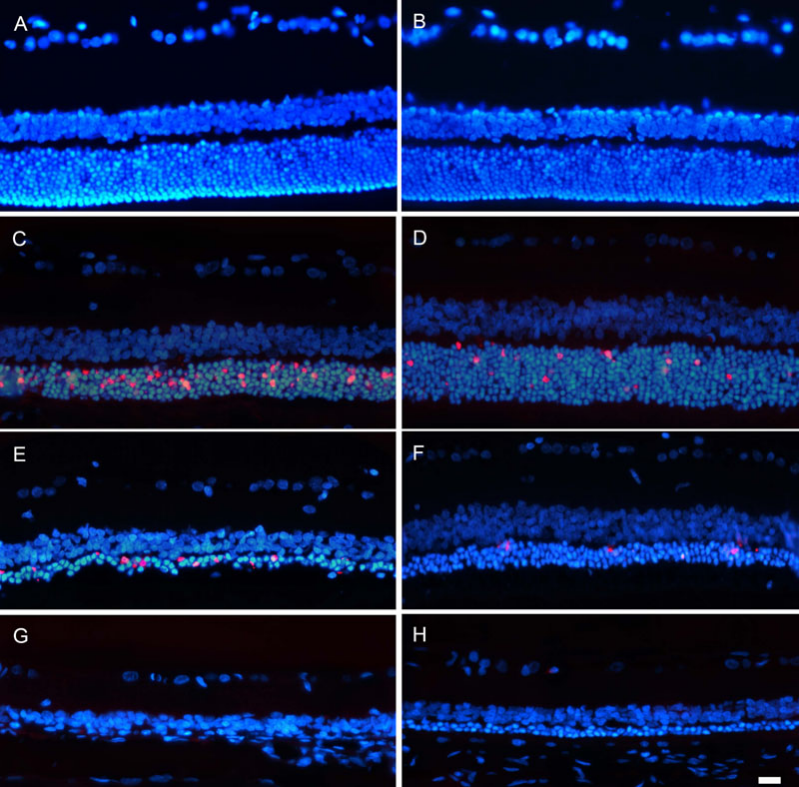
Light exposure induces apoptotic photoreceptor death. Photomicrographs of terminal deoxynucleotidyl transferase dUTP nick end labeling (TUNEL)-positive nuclei (red signal) in cross-sections from the mid-dorsal (left column) and midventral (right column) retina of control animals (**A**, **B**) and of light-exposed animals processed 0 h (**C**, **D**), 7 days (**E**, **F**), and 1 month (**G**, **H**) after light exposure (ALE). All retinal nuclei have been counterstained with 4’,6-diamidino-2-phenylindole (DAPI; blue). Immediately after ALE, TUNEL-positive nuclei were very abundant in the mid-dorsal retina (**C**), while in the ventral retina, only a few scattered TUNEL-positive photoreceptor nuclei were observed (**D**). Seven days ALE, the number of TUNEL-positive photoreceptor nuclei decreased, but there were still more apoptotic photoreceptors in the dorsal (**E**) than in the ventral (**F**) retina. One month ALE (**G**, **H**), no TUNEL-positive photoreceptor nuclei were observed. The scale bar represents 100 µm.

To assess whether photoreceptor loss ALE was due to apoptosis, the TUNEL assay was performed in cross-sections of control and experimental retinas at increasing survival intervals ALE. In control retinas, no TUNEL-positive photoreceptor nuclei were observed (Figure 4A,B). TUNEL-positive nuclei were detected in the ONL of the animals processed at early times ALE, 0 and 7 days (Figure 4C-F), but not at 1 month (Figure 4G,H), in spite of the fact that according to the hematoxylin and eosin–stained sections photoreceptors continue to degenerate during at least 3 months ALE.

### Light-induced retinal ganglion cell axonal abnormalities

**Figure 5:**
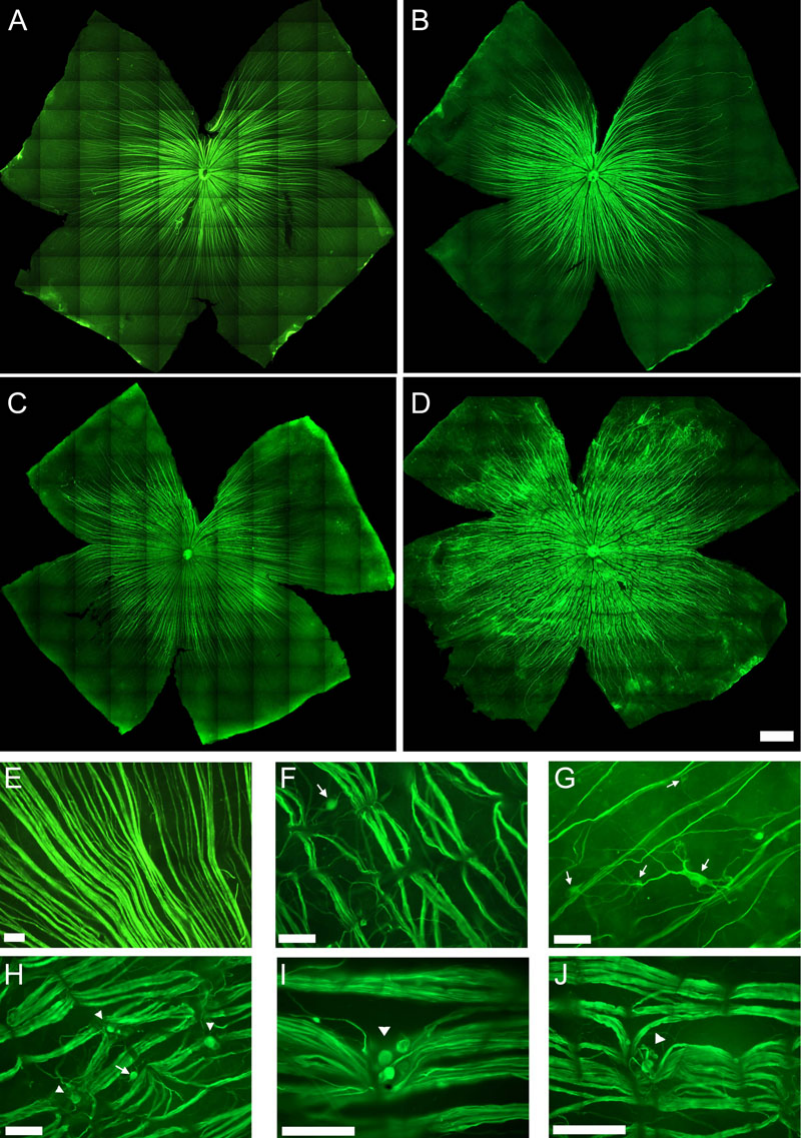
Retinal ganglion cell axonal abnormalities after light exposure. Photomontages of representative phosphorylated high molecular weight neurofilament subunit (pNFH) immunostained retinas from one control (**A**) and three light-exposed animals (**B-D**) processed 3 (**B**), 9 (**C**), and 12 (**D**) months after light exposure (ALE). The linear trajectory of the retinal ganglion cell (RGC) axons observed in the control retinas (**A**) becomes irregular in the retinas processed 3 or more (**B**-**D**) months ALE. These axonal abnormalities are more important and severe as the time ALE increases (compare **B** to **D**). **E**-**J**: Microphotographs of the optic nerve fiber layer in whole mount preparations of the retinas of one control animal (**E**) and five experimental animals processed 9 (**F**, **G**, **H**) or 12 (**I**, **J**) months ALE. In the experimental animals, axonal compressions by retinal vessels (**F**, **H**-**J**), axonal bulbs and meandering axons (arrowheads, **H-J**) and pNFH^+^ RGC somas (arrows, **F-H**) are observed. The scale bar represents 50 µm.

**Figure 6:**
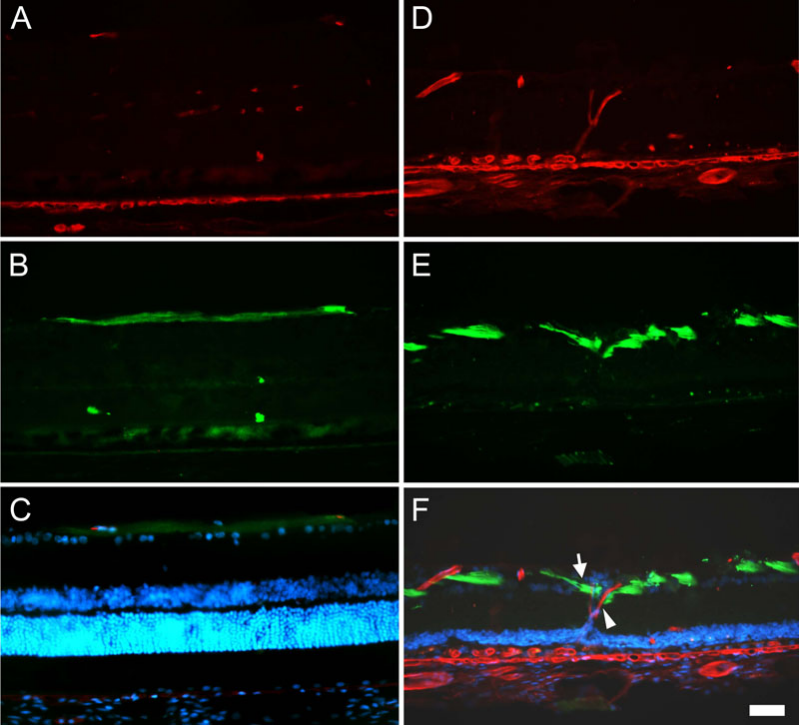
Retinal ganglion cell axons are compressed by displaced retinal vessels Representative retinal cross-sections from a control (**A-C**) and a photoexposed animal processed 12 months after light exposure (ALE; **D-F**) doubly immunoreacted to detect retinal vessels (red signal, **A** and **D**) and neurofilaments (green signal, **B** and **E**). In **C** and **F** are shown the corresponding coupled images and 4’,6-diamidino-2-phenylindole (DAPI) counterstaining. In control retinas, DAPI staining shows the typical layered structure of the retina, where retinal ganglion cell (RGC) axons run parallel to the retinal layers (**B**), above the RGC nuclei (**C**). ALE, however, the outer nuclear layer (ONL) has disappeared, DAPI positive nuclei are observed crossing vertically the inner plexiform layer (IPL; **F**) and the RGC axons are interrupted and dragged down (**E**, arrow) by retinal vessels vertically crossing the retina (**D**, arrowhead). The scale bar represents 100 µm.

In control retinas, pNFH^+^ axons showed a linear trajectory from the optic disc to the medial region of the retina (Figure 5A,E), as previously described [5,29]. This linear trajectory and expression pattern was maintained in the retinas processed early ALE. However, in the animals processed 3 months or more ALE, pNFH^+^ axons presented distorted nonlinear trajectories caused by regions of axonal strangulations (Figure 5D,F,H-J). These compressions appeared first in the dorsal retina but extended throughout the retina and became more frequent and severe with time (Figure 5D). By 6 months ALE, axonal bulbs and wandering axons could be seen above and below the compression points (Figure 5 H-J). These axonal strangulations were caused by the external displacement of vessels that cross the retinal nerve fiber layer (Figure 6, see also Figure 3Q-T) and drag the RGC axons, causing their strangulation and eventual severing.

In addition to these axonal aberrations, 3 or more months ALE it was also observed that some RGC bodies and their proximal dendrites became pNFH^+^ (Figure 5F-H), an abnormal expression pattern that we have described as a hallmark of neuronal degeneration [29].

### Delayed light-induced retinal ganglion cell loss

**Figure 7:**
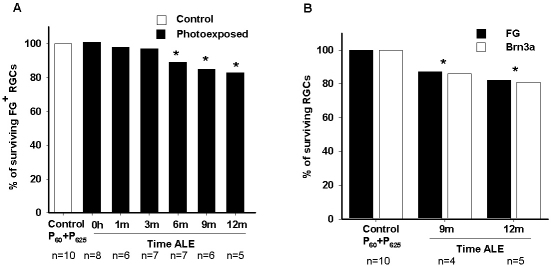
Percentage of retinal ganglion cell loss after light exposure. **A**: Graph showing the percentage of surviving fluorogold (FG)-traced retinal ganglion cells (RGCs) at increasing times (h represents hours, m represents months) after light exposure (ALE). The loss of RGCs is first significant 6 months ALE. **B**: Graph showing the percentage of surviving FG- or Brn3a-positive RGCs 9 and 12 months ALE. With both markers, it is observed that at these time points, there is a significant diminution of the RGC population. In both graphs, the percentage of labeled cells in control animals (100%; P60+P365) represents the mean number of labeled cells in young (P60) and old (P365) animals.

**Figure 8:**
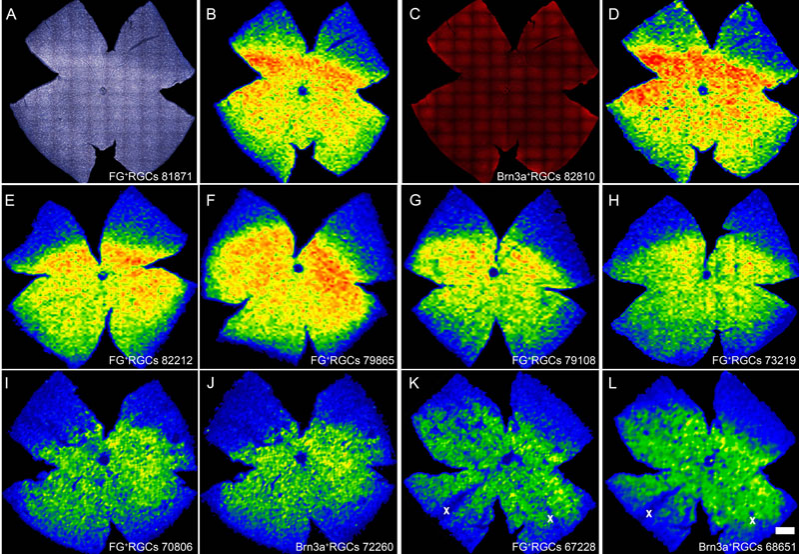
Topography of retinal ganglion cell loss after light exposure. **A** and **C**: Photomontages of a representative control retina showing retinal ganglion cells (RGCs) identified by fluorogold (FG) tracing (**A**) or Brn3a immunodetection (**C**). **B** and **D**: Corresponding isodensity maps showing the spatial distribution of FG- or Brn3a-positive RGCs. These maps are filled contour plots generated by assigning a color code to each one of the subdivisions of each individual frame according to its RGC density value within a color-scale range that goes from 0 (purple) to 3,500 or higher (red) RGCs/mm^2^. With both markers, it is observed that in control retinas, RGCs are densest in the dorsal pole, along the nasotemporal axis (**A**-**D**). **E** to **L**: Isodensity maps obtained from representative photoexposed retinas processed at increasing times ALE: 0 h (**E**), 1 month (**F**), 3 months (**G**), 6 months (**H**), 9 months (**I**, **J** generated from the same retina where RGCs were doubly identified by FG-tracing [**I**] and Brn3a detection [**J**]), and 12 months (**K**, **L** generated from the same retina where RGCs were doubly identified by FG-tracing (**K**) and Brn3a detection (**L**)). RGC loss is observed at 6 months ALE (**H**), as warm colors (red-oranges) are replaced by cooler ones (yellow-green-blues). At 9 (**I**, **J**) and 12 months (**K**, **L**) ALE, yellows and oranges have almost disappeared from the maps, indicating that RGC loss has gone further; this is observed to the same amount whether RGCs are identified by FG tracing (**I**, **K**) or Brn3a expression (**J**, **L**). The wedge-shaped areas of RGC loss have been marked with an X in **K-L**. The bottom of each map shows the number of RGCs counted in the retina wherefrom the map has been generated. The superior pole is at 12 o’clock. The scale bar represents 1 mm.

The whole population of FG-traced RGCs was counted in the right and left experimental retinas processed at increasing times ALE (Table 2, Figure 7, Figure 8). There was no significant difference (Mann–Whitney test) between the number of RGCs counted in the left and right retinas within each time point, at any period analyzed. The number of FG-traced RGCs started to decrease at 1 and 3 months ALE; however, it was not until 6 months that the RGC population had significantly diminished compared to control rats (P60 or P365, Mann–Whitney test, p<0.001; Figure 7A, Figure 8A,E-H, Table 2). From 6 until 12 months ALE, the percentage of RGC loss progressed further, though not significantly, and so in this period ALE it represented between 10 and 17% of the total RGC population (Figure 7A, Figure 8A,E-H; Table 2).

**Table 2:** Quantification of the total population of RGCs in control and experimental retinas at different periods after light exposure.

** **	**Control P60**				**Control P625**			
** **	**FG**		**Brn3a**		**FG**		**Brn3a**	
**Retina**	**Left**	**Right**	**Left**	**Right**	**Left**	**Right**	**Left**	**Right**
1	79,090	82,653	78,732	87,628	80,535	86,019	85,923	80,348
2	77,567	74,237	90,197	93,896	82,306	83,783	82,264	86,695
3	76,965	79,478	87,835	81,566	77,599	76,522	73,943	84,896
4	82,944	84,076	90,263	82,157	82,363	75,536	85,813	84,025
5	86,150	86,657	83,680	90,021	87,124	86,927	82,157	82,043
Mean±SD	80,534±3,492	81,420±4,274	86,141±4,410	87,053±4,691	81,985±3,098	81,757±4,798	82,020±4,871	83,601±2,471
RE & LE Mean±SD	80,981±3,928		86,597±4,575		81,871±4,040		82,810±3,544	
	0 h ALE		1 month ALE		3 months ALE		6 months ALE	
	FG		FG		FG		FG	
Retina	Left	Right	Left	Right	Left	Right	Left	Right
1	77,599	83,222	77,056	76,898	75,042	73,903	69,502	68,920
2	72,212	81,476	78,765	83,769	78,143	75,253	76,384	69,076
3	79,741	81,811	78,222	81,657	85,926	86,537	74,067	65,018
4	79,566	82,836	75,421	78,090	75,422	78,678	67,569	59,964
5	84,260	70,931	81,745	81,662	85,240	83,579	85,791	85,077
6	85,777	86,247	82,745	82,350	78,962	81,399	83,231	74,394
7	88,858	81,813			72,119	77,312	70,240	75,843
8	88,846	90,211						
Mean±SD	82,107±5,470	82,318±5,120	78,992±2,543	80,737±2,423	78,693±4,822	79,523±4,219	75,255±6,487	71,184±7,545
RE & LE Mean±SD	82,212±5,298		79,865±2,633		79,108±4,549		73,219±7,324 §	
	9 months ALE				12 months ALE			
	FG		Brn3a		FG		Brn3a	
Retina	Left	Right	Left	Right	Left	Right	Left	Right
1	79,691	74,467	#	#	76,939	81,085	#	#
2	79,849	77,367	#	#	73,287	72,567	#	#
3	63,045	67,771	#	#	49,278	58,418	#	#
4	50,506	49,366	#	#	70,271	61,707	#	#
5	79,281	73,755	#	#	70,240	64,704	#	#
6	64,954	66,632	#	#	64,199	71,006	67,899	69,783
7	71,375	73,308	72,185	73,010	66,378	70,817	64,725	67,955
8	71,112	71,992	73,506	72,970	54,433	70,131	67,779	70,999
9	70,420	69,746	73,952	69,119	71,656	63,259	61,138	76,592
10	65,203	73,296	69,397	73,944	70,404	70,004	69,647	69,997
Mean±SD	69,543±9,159	69,770±7,857	72,260±2,050	72,261±2,142	66,708±8,641	68,369±6,506	66,237±3,001	71,065±2,932
RE & LE Mean±SD	69,656±8,306§		72,260±1,941§		67,539±7,493§		68,651±2,413§	

In all retinas, RGCs were identified by retrograde tracing with fluorogold (FG). In addition, RGCs present in control retinas and in some retinas analyzed 9 and 12 months ALE were identified as well by Brn3a immunodetection. The whole population of FG or Brn3a positive RGCs was automatically quantified. At the bottom of each group is shown the mean ± the standard deviation (SD) of RGCs counted in the left or right retinas. Because there was not a significant difference in the number of RGCs between right and left retinas within each group, is also shown the mean±SD of RGCs present in the right and left eyes of each group. P60 and P365 refer to control animals of 60 and 365 days of age, respectively. RE: right eye, LE: left eye. ALE: after light exposure. §: Statistically significant when compared to their respective control values (Brn3a or FG) (Mann–Whitney test p<0.001)

Because after light exposure, RGC axons are strangulated by retinal vessels, it is possible that the decrease of FG-traced RGCs reflected an impairment in the transport of FG to the soma rather than actual RGC death. To fully address this issue, RGCs in control retinas and at 9 and 12 months ALE were detected, as well, by their expression of Brn3a. In these retinas, FG- and Brn3a-positive RGCs were quantified (Table 2, Figure 7B, Figure 8). These data show that there was a decrease of Brn3a-positive RGCs that paralleled the diminution of FG-traced RGCs, indicating that, at least at these times ALE, there is RGC death.

### Topography of retinal ganglion cell loss

Based on the quantification data, isodensity maps showing the spatial distribution of FG or Brn3a-positive RGCs in control and in experimental retinas were generated. During the first 6 months ALE, RGC distribution resembled that seen in control animals (Figure 8). In agreement with the quantification data, 6 months or more ALE there was a tendency for the highest RGC densities (represented by warm colors, red-oranges) to disappear, reflecting RGC loss. At 9 and 12 months ALE, yellows and oranges (high-medium densities) were being replaced by green-blues (lower densities), indicating a further RGC loss; as mentioned above, however, this decrease was not statistically significant. Importantly, RGC loss was observed whether RGCs were identified by tracing or Brn3a expression, and the topographic maps of FG-traced or Brn3a positive cells were very similar, indicating that both methods were similarly reliable.

RGC loss was not evenly spread throughout the retina, because some retinal regions were more affected than others (Figure 8H-L), and some retinas showed a few wedge-shaped areas of cell loss (Figure 8K,L) by 12 months ALE. In these areas, both FG-traced and Brn3a-positive RGCs were absent, indicating that they were the result of sectorial RGC death.

## Discussion

In this work, we studied the effects of light exposure on the albino rat retina and demonstrated that it causes, primarily, a rapid photoreceptor degeneration whose severity depends on the light source, the region of the retina, and pupil dilation. After photoreceptor death, there is a progressive retinal degeneration that affects all retinal layers, causing thinning of the outer plexiform layer and of the INL, and displacement of the inner retinal vessels that cross the nerve fiber layer, dragging the RGC axons. Consequently, RGC axons start to show an abnormal morphology and expression of pNFH. Finally, due to vessel traction of their axons, there is an impairment of the axonal transport, axonal interruption, and the RGCs die. We have thus documented that in the albino rat, light exposure causes the same degenerative events that we have already observed in other inherited [2,3] or light-induced [6] (Table 3) models of retinal degeneration. However, we also documented that there are important differences between these models that illustrate that both light pigmentation and the etiology of photoreceptor degeneration influence retinal remodelling after photoreceptor degeneration.

**Table 3:** Differences between pigmented and albino rats after light-induced retinal degeneration.

**Pigmented**	**Albino**
**1. Pupil dilation**	
Necessary for phototoxicity.	Not necessary, but increases light toxicity at early periods.
**2. Light-sensitive “arciform area” in the dorsal retina.**	
Blood retinal barrier breakdown almost always present in this area.	Blood retinal barrier breakdown present in this area only in 41% of the retinas.
Longer duration of HRP leakage (up to one month).	Shorter duration of HRP leakage (up to 7 days).
Retinal degeneration in this area much more severe: a very thin retina containing a few cells and the retinal nerve fiber layer remained 1 year ALE.	Photoreceptor degeneration starts and is more severe in this area, but only at early periods ALE
**3. Diffuse photoreceptor degeneration in areas other than the light sensitive “arciform” area.**	
Less severe than in albino rats.	More severe than in pigmented rats.
**4. Axonal compression by retinal vessels.**	
Two types of axonal compressions: a “localized” in the “arciform area” and a “diffuse” in the rest of the retina.	Only is observed the “diffuse” axonal compression
Starts earlier and progresses faster than in albino rats: it is observed 21 days ALE in the light sensitive “arciform” area (causes significant RGC death in the superior retina, see below) and by 4 months ALE has extended throughout the whole retina. This late “diffuse” form of axonal compression is more prevalent in the peripapillary ventral retina.	Starts later, at around 3 months and is “diffuse,” although at early times (3–9 months) is more prevalent in the dorsal retina.
**5. Retinal Ganglion cell death.**	
Following the two types of the axonal compressions, there are two types of RGC loss: a “localized” form peripheral to the “arciform area” and a “diffuse” form that sometimes is sectorial (wedge-shaped sectors).	RGC death is “diffuse” although at late times ALE it is observed a sectorial (wedge-shaped sectors) loss.
Is more abundant in the superior retina, due to the axonal compressions in the light sensitive “arciform” area. Wedge-shaped sectors lacking RGCs can be seen 9 or more months ALE in the ventral retina.	Is diffuse and significant 6 months ALE. Wedge-shaped areas lacking RGCs can be seen at12 months ALE mainly in the ventral retina.
**6. Subretinal vascular complexes.**	
Were seen always in the areas where photoreceptor degeneration was almost complete	
Start earlier and are more abundant: could be seen from 7 days ALE in the “arciform area,” from 3 months in the peripapillary and all throughout the ventral retina at 9 months.	First seen at around 6 months in the dorsal retina and extended throughout the retina with time ALE.

Light intensity was 3000 lx both for pigmented and albino animals. The duration of light exposure was 72 h in the pigmented rats and 48 h in albino rats. The duration of light exposure was reduced because in preliminary experiments we observed that 72 h of light exposure had devastating effects on the albino rat retina (data not shown). Data from pigmented animals are from: Marco-Gomariz et al., J Comp Neurol 2006 [6]. HRP: Horse-radish peroxidase. ALE: After Light Exposure.

The degree of retinal damage by light exposure depends, among other factors, on eye pigmentation [12,35-37] and light wavelength [38,39]. In opposition to pigmented rats [6,7,12,35], in albino rats pupil dilation is not necessary to induce retinal degeneration (Table 3), which supports the dependence of light damage on ocular pigmentation [12,35-37]. Even so, our data show that a maintained mydriasis during the photoexposition period induces a quicker degeneration than the one induced under miotic conditions. However, this difference is only observed up to a month after light exposure.

In this work, damage was induced by photoexposing the animals to white fluorescent light, which contains all wavelengths. Because in our previous study, we used only linear bulbs and found that light damage was more severe in the dorsal retina [6] (Table 3), in this study, we used two types of cold fluorescent bulbs—linear bulbs situated in the ceiling and circular bulbs situated around the transparent cages—to observe whether the location of the light source influences the location of the retinal damage. Although in both instances, light intensity within the cages was similar (3,000±100 lux), and the inflicted damage was comparable at 3 months ALE, this was more severe at earlier times when circular bulbs were used. One possible explanation for this is that when circular bulbs are used, the animals receive light from all directions. However, we did not find, as we were expecting, any differences in the location of the retinal damage between the two different light sources.

Very early ALE, two pathological signs are observed: vascular leakage and photoreceptor death. In pigmented rats, light exposure causes two separate retinal degenerative events that may relate to differential light exposure across the retina: an early arciform area of degeneration in the superotemporal retina and a delayed degeneration in the central and ventral retina [6]. The arciform area, observed by vascular leakage of HRP, lasted up to a month ALE, and was the most severely affected region [6] (Table 3). In the albino retina, vascular leakage in the arciform area was observed only during the first week ALE just in 22 of the 54 retinas analyzed, indicating that HRP leakage within this area is less prevalent in albino than in pigmented animals (Table 3). Thus, the amount and duration of vascular leakage may depend, at least in part, on the melanin content of the eye. It is worth noting that, in spite of this lesser frequency and duration of vascular leakage, the degeneration in the albino strain, as in the pigmented one, started and was more severe in the arciform area (Table 3). Interestingly, this area is located in the “photosensitive area” described in the superior retina in normal rats [1,12-15,40,41].

Melanin content also has an effect on the degree of photoreceptor death. Photoexposition of pigmented rats to the same light intensity as the albino animals in this study (3,000 lux), but during a longer period (72 h), induced photoreceptor loss; importantly, however, this only occurred in the dilated eyes [6]. Moreover, the amount of photoreceptor death was somewhat smaller than in the albino rats: The ONL decreased to 0–1 nuclei thick, except in the arciform area where, with time, most retinal layers disappeared [6] (Table 1 and Table 3). In the albino rat, photoreceptor degeneration is more dramatic, and at 3 months ALE the ONL has disappeared, independently of pupil dilation (Table 1 and Table 3). Curiously, the degeneration in the arciform area did not progress to the point seen in pigmented retinas. Therefore, albino rats are more sensitive to light because, in dilated and nondilated eyes, a smaller period of exposure produces the same amount of cell death as the degeneration observed in pigmented rats subjected to mydriasis and longer exposure time. However, in contrast, albino rats show less long-term degeneration in the arciform area than the pigmented ones (Table 1 and Table 3). We could speculate that this is due to a greater toxicity of light in this area in pigmented animals or to factors other than ocular pigmentation, as there are many other differences between albino and pigmented animals.

In this study in albino animals, from the first day ALE, the components of the ERG that correspond to the function of the outer and inner retina were almost completely abolished. Similar results have been reported by other authors using similar methods [42,43]. These data correlate well with the early photoreceptor loss observed in hematoxylin and eosin–stained oriented cross-sections, which was more severe in the superior retina and in the dilated eyes and with the TUNEL-positive nuclei observed at 0 h and 7 days ALE.

Light induces photoreceptor death by apoptosis [44]. Although photoreceptor death in hematoxylin and eosin–stained sections was observed during at least 3 months ALE, TUNEL-positive nuclei were only found in the retinas processed from 0 h or 1 week ALE. This apparent discrepancy may be explained by the fact that the bulk of cell death occurs between 0 and 7 days ALE, and because apoptosis is a very rapid event that takes only hours to complete, very few cells die at a given point at later stages, and thus are hard to find. On the other hand, it may be that not all the photoreceptors die by apoptosis, as has been suggested [45,46; see below].

From 3 months ALE, there is an affectation of the inner retina that was first observed by the abnormal expression of pNFH. In these retinas, the axonal pNFH signal reached the retinal periphery and was also detected in some RGC somas and dendrites, mimicking the aberrant expression pattern, the hallmark of RGC degeneration, described after light toxicity in pigmented animals [6], optic nerve injury [29], and ocular hypertension [22,47]. Thus, at 3 months ALE, RGCs started to show signs of degeneration that ended 3 months later in a significant decrease of this neuronal population.

What caused the death of RGCs? In flat-mounted retinas, it was observed that RGC axons presented distorted nonlinear trajectories caused by strangulations. These axonal strangulations were more severe and frequent with time ALE and resembled those described in the retinas of rats and mice with inherited retinal degenerations [2-5,28] and in pigmented rats ALE [6]. These strangulations are caused by displaced vessels that, after crossing the optic fiber layer, run deep to supply subretinal vascular complexes and in doing so, traction the RGC axons. These vessels ran vertically from the inner retinal vascular plexus and are connected to subretinal vascular formations that belong to the outer plexus of the retinal circulation [4,6], and have been observed as well, though they were less extensive, in RCS-p+, P23H, and pigmented light-exposed rats [3-6].

Six months or more ALE, when RGC degeneration was significant, there were axonal bulbs and wandering axons above and below the vessel strangulations, suggesting that the axonal transport has been interrupted, causing an axotomy-like insult; this is further supported by the abovementioned aberrant expression of pNFH and later RGC death.

Because RGC death in this model was triggered by axonal interruption, it was possible that the decrease of FG^+^ RGCs was due to a deterioration of axonal transport rather than actual RGC death. To investigate this possibility, RGCs in control and at 9 and 12 months ALE were identified by retrograde axonal tracing and Brn3a expression. The number of surviving Brn3a^+^ RGCs matched that of FG^+^ RGCs, demonstrating that phototoxicity induces a delayed degeneration and death of RGCs. The parallelism between the numbers of FG-labeled and Brn3a-labeled cells in these retinas suggests that RGC death caused by axonal compression is a very rapid event.

RGC degeneration is observed when photoreceptor loss is almost complete, in agreement with other models of either induced or inherited photoreceptor degeneration, supporting the hypothesis that it is due to retinal remodeling after photoreceptor degeneration [2,3,5,6]. Interestingly, in the albino strain, photoreceptor death was equally dramatic in dilated and nondilated eyes from 3 months onwards, and so, entailed the same level of RGC loss in both eyes, independently of pupil dilation (Table 3). Isodensity maps showed that in albino animals, RGC death was distributed more or less evenly throughout the retina. This pattern of cell death is thus different than that observed in pigmented animals ALE because, in this strain, RGC death was mainly localized in the superior retina, peripheral to the arciform area of maximal degeneration [6] (Table 3). On the other hand, both albino animals and pigmented animals ALE [6] (Table 3) and dystrophic RCSp+ rats [2,3] show wedge-shaped sectors lacking RGCs. This is consequent with the proposed mechanism of axotomy-induced RGC loss: Because vessels sever bundles of axons and not individual axons, RGC loss is observed in pie-shaped sectors. In this study, we documented using two different methods—FG tracing and Brn3a expression—that the diminution in RGC densities after photoreceptor degeneration is due to RGC death. RGC death has also been shown to a large extent in retinitis pigmentosa [8-11], but it remains to be shown whether this is due to the same remodeling mechanism.

In conclusion, these analyses demonstrate that light-induced toxicity in albino rats causes apoptotic photoreceptor death, which appears earlier in an arciform area located in the mid-dorsal retina, but progresses with time, spanning the whole retina. Secondary to photoreceptor degeneration and due to axonal compression by displaced blood vessels, RGCs are also affected and at long survival times ALE, their number diminishes. Furthermore, we have also demonstrated that this lesion abolishes permanently and almost completely all the waves associated with the ERG response. Because these degenerative events have been described before but occur differently in pigmented animals with inherited retinal degenerations or ALE [3-6,28], they deserve further investigation to understand the influence of eye pigmentation and the etiology of photoreceptor degeneration in retinal remodelling. Finally, we would like to stress that, strategies aimed to protect the retina in light-induced and inherited retinal degenerations should be implemented early, before the RGCs are affected.
